# Disclosure of gynecological cancer diagnosis: a mixed-methods study from an Islamic cultural context

**DOI:** 10.1186/s43046-026-00386-3

**Published:** 2026-07-21

**Authors:** Israa Malli, Hatim Al-Jifree, Farah Bakhamees, Loujen Alamoudi, Ola Bahadur

**Affiliations:** 1 College of Medicine, King Saud bin Abdulaziz University for Health Sciences, Jeddah, Saudi Arabia; 2 King Abdullah International Medical Research Center, Jeddah, Saudi Arabia; 3 National Guard Health Affairs, King Saud bin Abdul-Aziz University for Health Sciences, Jeddah, Saudi Arabia

**Keywords:** Breaking bad news, Gynecological cancer, Disclosure, Saudi Arabia, Patient autonomy, Cultural ethics

## Abstract

**Background:**

Delivering devastating news of cancer is a universal challenge, but cultural and religious contexts impact disclosure. In Saudi Arabia, family engagement frequently influences communication, yet little is known about stakeholders’ perspectives on gynecological cancer.

**Methods:**

This mixed-methods study used validated questionnaires and semi-structured interviews.

**Results:**

A total of 737 participants were included (54.1% public, 18.5% physicians, 16.0% relatives, and 11.4% patients); the majority were female (67.8%) and Saudi citizens (81.9%), with a median age of 38 years.

**Results:**

Overall, 90.5% of participants preferred that a cancer diagnosis be disclosed to the patient rather than withheld. However, only 25–36% supported disclosing a bad prognosis, with physicians being the least supportive (6.7%, *p* < 0.0001). Most groups supported the use of the term “chemotherapy” (88–98%). 94% of clinicians favored disclosing treatment failure, but only 61% of patients and 53% of the public agreed (*p* < 0.0001). More than half of patients (51.5%) and three-quarters of clinicians (73.1%) were opposed to concealing diagnoses at the request of the family. Patients greatly preferred engagement in treatment (66.3%) and end-of-life talks (80.7%), whereas physicians showed less support. In terms of communication dynamics, most patients preferred that the head of the medical team disclose directly to them, face-to-face, and framed with “Allah’s will.”

**Conclusions:**

Although patients, family, the public, and clinicians mostly supported direct disclosure, there were still disagreements over prognosis and end-of-life talks. These findings emphasize the importance of culturally sensitive, ethically based communication training to enhance patient-centered treatment in gynecological oncology.

## Introduction

Delivering bad news remains a significant challenge for physicians across medical specialties, with some facing greater pressure than others. Oncologists, in particular, are at the forefront of this challenge, as they frequently communicate life-altering cancer diagnoses [1]. Buckman defined bad news as information that “dramatically changes or negates the patient’s vision of the future” [2]. A cancer diagnosis exemplifies this definition, often generating profound psychological distress for both patients and their families due to its life-threatening nature and its adverse impact on health and quality of life [3].

Gynecological cancers collectively contribute substantially to the cancer burden among women in Saudi Arabia. Recent national data indicate that gynecological malignancies account for a significant proportion of cancer diagnoses and related mortality, underscoring the need for effective prevention, early detection, and education strategies within the local healthcare context [4]. In Saudi Arabia, cervical cancer remains relatively rare but is still a critical public health concern [5, 6]. The country records roughly 358 new diagnoses annually, resulting in approximately 179 deaths from the disease each year [7, 8]. Additionally, an analysis of national registry data revealed that while the incidence of invasive cervical cancer remains low, the proportion of cases detected at localized stage has notably increased up to 40% in 2023 [6, 8].

Cancer incidence and death rates continue to climb worldwide. As a result, once diagnostic results are available, clinicians must decide how to best reveal the diagnosis and convey the results, treatment choices, and prognosis to their patients [9]. Given the gravity of cancer, professionals must also respect patients’ rights and wishes, ensuring that disclosure occurs via effective, patient-centered communication [3]. Evidence shows that this method improves treatment adherence, medication compliance, and overall patient satisfaction [1].

Although international ethical standards outline best practices for delivering difficult diagnoses, the process is deeply influenced by cultural norms and accepted customs within each community [10, 11]. In Saudi Arabia, a Middle Eastern country with an Islamic cultural foundation, the family plays a central role in decision-making due to deeply rooted values and religious guidance [12, 13]. Two studies conducted in Saudi Arabia explored patients’ perceptions of cancer disclosure to support the delivery of serious diagnoses in line with patients’ needs and preferences [14, 15]. These studies reported that families were often the first to be informed of the patient’s condition and frequently assumed a dominant role in decisions regarding treatment options [16]. Nevertheless, many patients with cancer expressed a strong preference to be directly informed of their diagnosis and to actively participate in decisions about their care [14, 15].

Islamic ethics emphasize truthful and compassionate communication with patients. The Quran encourages believers to “speak words of appropriate justice” (Quran 33:70) and to “speak to him with gentle speech” (Quran 20:44). These principles may influence expectations regarding the disclosure of serious illnesses, including gynecological cancer. Despite the rising literature on breaking bad news in oncology, few studies particularly investigate gynecological cancer in Saudi Arabia’s distinct cultural and religious setting. The purpose of this study was to investigate patients’, physicians’, and relatives’ perceptions and experiences with disclosing gynecological cancer diagnoses.

## Materials and methods

### Study setting, design, and duration

This mixed-methods study explored perceptions, preferences, and experiences related to the disclosure of gynecological cancer diagnoses among three stakeholder groups: patients and their relatives, physicians, and members of the public. The study was conducted at King Abdulaziz Medical City (KAMC) in Jeddah, Saudi Arabia, and at four major shopping malls across the city’s geographic regions (north, south, east, and west), between May 2019 and August 2021.

### Sample size and sampling technique

The sample size was calculated using the Raosoft Sample Size Calculator with a 95% confidence level, a 5% margin of error, and an expected response distribution of 50% [17]. Calculations accounted for multiple populations, including the number of obstetrics and gynecology and oncology physicians at KAMC, the population of Jeddah (3.431 million), the number of gynecologic oncology cases treated at KAMC, and the reported incidence of gynecological malignancies in Saudi Arabia [18]. The estimated sample sizes were 49 obstetrics and gynecology physicians, 77 oncology physicians, 77 gynecologic oncology patients, 77 relatives (using a 1:1 patient-to-relative ratio), and 385 members of the public, yielding a total target sample of 665 participants.

Patients and relatives were recruited from gynecologic oncology outpatient clinics and inpatient wards at KAMC. Eligible patients included women with a confirmed diagnosis of gynecological cancer at any stage of care, including newly diagnosed patients, those undergoing active treatment, and patients in early survivorship. The study did not restrict inclusion to a specific time point after diagnosis, thereby capturing a broad range of lived experiences related to diagnostic disclosure. Relatives accompanying patients during clinic visits or hospitalization were invited to participate; relatives younger than 18 years were excluded.

Physicians were recruited from KAMC and included consultants, fellows, specialists, residents, and registrars involved in the care of patients with gynecological malignancies, including obstetrics and gynecology, gynecologic oncology, medical oncology, radiation oncology, hematology, bone marrow transplantation, and palliative care. Members of the general public were recruited from shopping malls to capture community perspectives on cancer disclosure in individuals without a personal cancer diagnosis. Shopping malls were selected as recruitment sites because they provide access to a diverse cross-section of the population across age, gender, education level, and socioeconomic background. Public participants were approached by trained research assistants and invited to participate voluntarily. Individuals younger than 18 years were excluded.

The qualitative component used purposive sampling to recruit information-rich participants from patients, relatives, and physicians. Maximum variation sampling and deviant case sampling were applied to capture diverse perspectives, with recruitment continuing until thematic saturation was achieved [19]. To address the differing perspectives of participants with cancer experience versus those without, analyses were conducted separately by group. Comparisons between groups were interpreted cautiously and framed as differences in perspectives rather than direct equivalence of experience. The inclusion of both clinical stakeholders and community members was intentional, allowing the study to contrast experiential views with societal beliefs surrounding cancer disclosure within the same cultural context. In addition, participants from the general public and healthcare provider groups were asked to report any personal or family history of cancer. Accordingly, the variable type of cancer reflects cancer exposure (self or family history), rather than indicating that all participants were cancer patients.

### Study instruments and data collection

A paper-based questionnaire was utilized for the quantitative data collection. Two forms of the questionnaire were offered: version A (for patients, relatives, and the public) and version B (for physicians). The questionnaire, available in both English and Arabic, had previously been tested for validity and reliability in similar cultural settings [14, 20]. The questionnaire consisted of three main sections. The first page collected demographic information. The second section explored cancer-related exposures and perspectives, including questions about the type of cancer exposure, the information patients should receive, and a series of 5-point Likert-scale items assessing the degree of patient involvement in treatment-related discussions. The third section assessed preferences for disclosure, with questions regarding the preferred methods and settings for receiving bad news.

In addition to the questionnaire, participants in the qualitative arm participated in individual open-ended interviews administered by data collectors. For the qualitative data, all interviews were transcribed verbatim using Microsoft Word in the original language of the interview. Transcripts conducted in Arabic were subsequently translated into English in separate files to maintain accuracy and ensure comparability. Qualitative analysis commenced immediately after data collection. Two researchers independently reviewed each transcript, coding text segments and identifying emerging themes. After every three interviews, the researchers met to compare codes, reconcile differences, and refine the thematic framework. Inter-coder reliability was assessed by comparing independent coding results, achieving a Cohen’s kappa coefficient greater than 0.80, indicating strong agreement. Themes were then organized into broader categories and supported by illustrative quotations from participants [21].

### Statistical analysis

For quantitative data, responses were recorded on data collection sheets, coded, uploaded to Microsoft Excel, and then exported to JMP statistical software (version 15, SAS Institute Inc., Cary, NC, USA) for analysis. Non-parametric variables were presented as medians with interquartile ranges (IQRs), and normally distributed continuous variables were summarized as means with standard deviations (SDs). For continuous variables, data were compared using the unpaired Mann-Whitney U test for inferential analyses. Fisher’s exact test was used to compare categorical variables. P-values less than 0.05 were regarded as statistically significant.

### Ethical approval and informed consent

#### Ethical approval

for this study was obtained from the Institutional Review Board (IRB) of KAMC (approval memo no. SP19/109/J). For the quantitative arm, participants provided written consent, whereas for the qualitative arm, informed consent was obtained prior to interviews. To ensure confidentiality, all personally identifiable information was omitted, and the data were used exclusively for research purposes during the study period.

## Results

### Demographic information and participants’ characterization

A total of 737 participants were included in the study and stratified into three groups: cancer patients (*n* = 203), healthcare providers (*n* = 135), and members of the general public (*n* = 405). Overall, 68.1% were female (*n* = 502) and 31.9% male (*n* = 241). The median age of participants was 38 years (IQR = 14). The largest age groups were 20–29 years (29.7%), and 6.8% were above 60 years. Most respondents were Saudi nationals (81.9%), and most resided in the Makkah Province (84.9%), particularly in Jeddah. Regarding marital status, more than half were married (55.3%), while educational achievement varied across groups. In terms of cancer exposure, 287 respondents (38.9%) reported gynecological cancer, 198 (26.9%) reported non-gynecological cancer, and 258 (35.0%) reported both exposures across their networks. Table 1 summarizes the participants’ demographic information.

**Table 1:** Demographic data and cancer exposure of the participants

Variables	Categories	Population		
		Cancer Patient 203 (27.32)	General Public 405 (54.51)	Healthcare Provider 135 (18.17)
Gender	Male	74 (9.96)	107 (14.40)	60 (8.08)
	Female	129 (17.36)	298 (40.11)	75 (10.09)
Age	Less than 20 years	4 (0.54)	50 (6.73)	0 (0.00)
	20–29 years	25 (3.36)	165 (22.21)	29 (3.90)
	30–39 years	44 (5.92)	90 (12.11)	50 (6.73)
	40–49 years	52 (7.00)	69 (9.29)	40 (5.38)
	20–59 years	41 (5.52)	21 (2.83)	13 (1.75)
	More than 60 years	37 (4.98)	10 (1.35)	3 (0.40)
Nationality	Saudi	198 (26.65)	318 (42.80)	88 (11.84)
	Non-Saudi	5 (0.67)	87 (11.71)	47 (6.33)
Residency	Makkah Province	143 (19.25)	353 (47.51)	135 (18.17)
	Madinah Province	31 (4.17)	9 (1.21)	0 (0.00)
	Riyadh Province	4 (0.54)	22 (2.96)	0 (0.00)
	Eastern Province	1 (0.13)	4 (0.54)	0 (0.00)
	Other Provinces	24 (3.23)	17 (2.29)	0 (0.00)
Marital Status	Married	131 (17.63)	184 (24.76)	96 (12.92)
	Single	39 (5.25)	200 (26.92)	36 (4.85)
	Widowed	26 (3.50)	5 (0.67)	1 (0.13)
	Divorced	7 (0.94)	16 (2.15)	2 (0.27)
Educational Level	Less than high school	65 (8.75)	14 (1.88)	0 (0.00)
	High school	45 (6.06)	84 (11.31)	0 (0.00)
	Undergraduate	5 (0.67)	101 (13.59)	0 (0.00)
	Post-graduate	88 (11.84)	206 (27.73)	135 (18.17)
Cancer exposure *	Gynecological	170 (22.88)	97 (13.06)	20 (2.69)
	Non-Gynecological	14 (1.88)	127 (17.09)	57 (7.67)
	Both	19 (2.56)	181 (24.36)	58 (7.81)

*Cancer exposure reflects the cancer history reported by participants (self and or family member), as applicable

### Participant’s preferences regarding breaking gynecological cancer information

Overall, a large majority (90.5%) preferred that patients be informed of their cancer diagnosis, with significant variation across groups: 99.3% of healthcare providers, 90.6% of the general public, and 84.2% of patients supported disclosure (*p* < 0.0001). Preferences for disclosing a poor or unfavorable prognosis were far less common, reported by 36.3% of patients, 25.3% of the public, and only 6.7% of providers (*p* < 0.0001). Regarding treatment, most respondents supported using the term “chemotherapy” when it was part of the treatment plan: 88.6% of patients, 84.8% of the public, and 97.8% of healthcare providers (*p* = 0.0003). Disclosure of treatment failure or ineffectiveness showed more disagreement: nearly all providers (94.0%) endorsed disclosure, compared with 61.4% of patients and 52.7% of the public (*p* < 0.0001). Regarding updates during the disease course, participants were asked to disclose clinically relevant changes, including disease progression or improvement, treatment modifications, prognosis updates, recurrence, and complications. Most participants supported disclosure of significant changes in *condition* (no significant difference, *p* = 0.3225) and general health updates (77.9% overall, *p* = 0.1897). However, disclosure of the unavailability of anti-cancer treatment options revealed strong contrasts: 88.8% of providers supported disclosure, compared with 68.8% of patients and 54.5% of the public (*p* < 0.0001). Subgroup analysis showed no statistically significant differences between Saudi and non-Saudi participants across the seven primary disclosure outcomes (all *p* > 0.05). Participants’ preferences on breaking bad news in gynecological cancer are summarized in Table 2.

**Table 2:** Participants’ preferences regarding breaking gynecological cancer information

Should the patient be informed about		Population			
		Cancer Patient 203 (27.32)	General Public 405 (54.51)	Healthcare Provider 135 (18.17)	*P- Value*
1. Their diagnosis of cancer	Yes	171 (84.24)	367 (90.62)	133 (99.25)	**< 0.0001**
	No	32 (15.76)	38 (9.38)	1 (0.75)	
2. Any possible poor or unfavorable prognosis	Yes	73 (36.32)	102 (25.31)	9 (6.72)	**< 0.0001**
	No	128 (63.68)	301 (74.69)	125 (93.28)	
3. The use of the term “chemotherapy” if it is part of the treatment plan	Yes	179 (88.61)	340 (84.79)	131 (97.76)	**0.0003**
	No	23 (11.39)	61 (15.21)	3 (2.24)	
4. The failure or ineffectiveness of treatment	Yes	124 (61.39)	213 (52.72)	126 (94.03)	**< 0.0001**
	No	78 (38.61)	191 (47.28)	8 (5.97)	
5. Every significant change in their condition or expected outcome	Yes	182 (90.10)	354 (88.06)	124 (92.54)	0.3225
	No	20 (9.90)	48 (11.94)	10 (7.46)	
6. All relevant health updates related to their condition	Yes	165 (81.68)	306 (75.74)	108 (80.60)	0.1897
	No	37 (18.32)	98 (24.26)	26 (19.40)	
7. The unavailability of specific anti-cancer treatment options	Yes	139 (68.81)	219 (54.48)	119 (88.81)	**< 0.0001**
	No	63 (31.19)	183 (45.52)	15 (11.19)	

### Participant’s views and beliefs regarding breaking gynecological cancer-related news

Table 3 presents participants’ views and beliefs on withholding information at the request of family members and on patient involvement in treatment and end-of-life discussions. A substantial proportion of respondents rejected withholding information from patients: 51.5% of cancer patients, 46.8% of the public, and 73.1% of healthcare providers disagreed with concealing information at relatives’ request (p < 0.0001). Regarding patient involvement in treatment discussions, the majority across all groups supported active participation. An agreement was expressed by 66.3% of patients, 76.5% of the public, and 84.3% of providers, with statistically significant variation between groups (p = 0.0003). Similarly, most respondents believed that patients should be involved in end-of-life discussions, though agreement levels varied: 80.7% of patients, 74.8% of the public, and 67.2% of providers endorsed inclusion (p = 0.0237). Overall, these findings highlight that while a clear majority across groups valued transparency and patient participation, healthcare providers were more likely than patients and the public to oppose withholding information, yet comparatively less supportive of patient involvement in end-of-life discussions.

**Table 3:** Participants’ views and beliefs regarding breaking gynecological cancer-related news

		Population			
		Cancer Patient 203 (27.32)	General Public 405 (54.51)	Healthcare Provider 135 (18.17)	*P- Value*
1. The Physician should Withhold Information from the Patient at the Request of a Family Member	Disagree	104 (51.49)	189 (46.78)	98 (73.13)	**< 0.0001**
	Neutral	11 (5.45)	101 (25.00)	21 (15.67)	
	Agree	87 (43.07)	114 (28.22)	15 (11.19)	
2. The Patient should be involved in Treatment Discussions	Disagree	50 (24.75)	54 (13.33)	12 (8.96)	**0.0003**
	Neutral	18 (8.91)	41 (10.12)	9 (6.72)	
	Agree	134 (66.34)	310 (76.54)	113 (84.33)	
3. The Patient should be Involved in End-of-life Discussions	Disagree	23 (11.39)	58 (14.36)	19 (14.18)	**0.0237**
	Neutral	16 (7.92)	44 (10.89)	25 (18.66)	
	Agree	163 (80.69)	302 (74.75)	90 (67.16)	

### Participants’ preferences on communication dynamics and delivery of cancer bad news

Analyzing participants’ references to communication dynamics and the delivery of bad cancer news revealed distinct preferences regarding how and by whom such news should be delivered. Across all groups, the patient was the preferred first recipient of bad news, followed by the spouse or a close relative (p = 0.025). Close relatives were also most frequently selected as the second recipient and as the person who should accompany the patient during disclosure (p < 0.0001). Regarding the preferred communicator, most respondents favored the head of the medical team, followed by a member of the medical team or, less commonly, a hospital social worker (p < 0.0001). The overwhelming majority preferred face-to-face communication (89.0%), with very few endorsing alternatives such as phone, email, or medical reports (p < 0.0001). As for the style of disclosure, most participants supported beginning with “Allah’s will,” followed by a structured introduction about the disease, while very few favored direct disclosure without introduction (p < 0.0001). After disclosure, the majority believed that the informer should stay with the patient to provide further information, whereas fewer supported leaving the patient alone temporarily or asking relatives for support (p < 0.0001). However, nationality was significantly associated with preferences regarding the person accompanying the patient during disclosure (p = 0.0005) and the preferred person to deliver the cancer diagnosis (p = 0.0103), whereas no significant differences were observed for the remaining communication preference variables. Table 4 presents participants’ references on communication dynamics and the delivery of bad cancer news.

**Table 4:** Participants’ references on communication dynamics and delivery of cancer bad news

Statements	Variables	Population			
		Cancer Patient 203 (27.32)	General Public 405 (54.51)	Healthcare Provider 135 (18.17)	*P- Value*
The first person to receive cancer bad news.	Patient	117 (15.81)	242 (32.70)	86 (11.62)	**0.0250**
	Spouse	29 (3.92)	45 (6.08)	19 (2.57)	
	Close relative	53 (7.16)	101 (13.65)	28 (3.78)	
	Friend	3 (0.41)	17 (2.30)	0 (0.00)	
The second person to receive cancer bad news.	Close relative	118 (16.01)	219 (29.72)	43 (5.83)	**< 0.0001**
	Friend	7 (0.95)	41 (5.56)	25 (3.39)	
	No One	21 (2.85)	40 (5.43)	0 (0.00)	
	Spouse	55 (7.46)	104 (14.11)	64 (8.68)	
The person accompanying the patient during the receival of cancer bad news.	Close relative	116 (15.72)	202 (27.37)	50 (6.78)	**< 0.0001**
	Friend	6 (0.81)	31 (4.20)	14 (1.90)	
	No One	29 (3.93)	69 (9.35)	0 (0.00)	
	Spouse	51 (6.91)	103 (13.96)	67 (9.08)	
The preferred person to deliver cancer bad news	Head of medical team	126 (17.10)	189 (25.64)	112 (15.20)	**< 0.0001**
	Member of medical team	21 (2.85)	49 (6.65)	17 (2.31)	
	Close family member	9 (1.22)	44 (5.97)	3 (0.41)	
	Hospital social worker	45 (6.11)	102 (13.84)	0 (0.00)	
	Friend	1 (0.14)	19 (2.58)	0 (0.00)	
The preferred method to receive cancer bad news	Face-to-Face	179 (24.25)	338 (45.80)	133 (18.02)	**< 0.0001**
	Phone conversation	9 (1.22)	7 (0.95)	1 (0.14)	
	E-mail	3 (0.41)	2 (0.27)	0 (0.00)	
	Medical report	11 (1.49)	55 (7.45)	0 (0.00)	
The best way of breaking cancer bad news	Directly without introduction	13 (1.78)	38 (5.19)	4 (0.55)	**< 0.0001**
	Start with Allah’s will	157 (21.45)	279 (38.11)	35 (4.78)	
	Start with introduction about the disease	32 (4.37)	86 (11.75)	88 (12.02)	
The thing that the informer should do after breaking cancer bad news	Ask one of the patient’s family or to come and support her	15 (2.03)	66 (8.92)	13 (1.76)	**< 0.0001**
	Leave the patient alone and do not come back	11 (1.49)	17 (2.30)	0 (0.00)	
	Leave the patient alone for a while	23 (3.11)	108 (14.59)	21 (2.84)	
	Stay and give more information about the disease	97 (13.11)	104 (14.05)	26 (3.51)	

## Discussion

This mixed-methods study highlights the complex dynamics surrounding the disclosure of gynecological cancer diagnoses in Saudi Arabia. The majority of participants (90.5%) preferred straightforward disclosure of a cancer diagnosis; significant disparities arose in more sensitive concerns such as prognosis, treatment failure, and the lack of specific treatment alternatives. These findings indicate both progress and ongoing obstacles in developing a culture of transparency in cancer communication in Saudi Arabia.

The inclusion of three distinct groups—patients and family, healthcare practitioners, and the general public—enhanced the mixed-methods approach by enabling comparison of viewpoints among stakeholders. Similar designs have been used in research from different Middle Eastern cultures, where family members frequently play an important role in medical decision-making [14, 22]. Healthcare professionals overwhelmingly supported diagnostic disclosure (99.3%), consistent with international ethical norms that emphasize patient autonomy [2]. However, clinicians (6.7%) were the least likely to support disclosing a bad prognosis, compared to 36.3% of patients and 25.3% of the general population. This hesitation may originate from clinicians’ worries about psychological harm to patients, as seen in research from China and Iran, where cultural and professional norms advocate safeguarding patients from painful information [23–25].

Interestingly, healthcare personnel significantly supported disclosure of treatment failure (94%), although less than two-thirds of patients (61.4%) and slightly more than half of the general public (52.7%) did. This disparity shows that doctors may regard honesty about treatment limitations as an ethical imperative, but patients and families may see it as a loss of hope. Similar tendencies have been observed in research on the Middle East, where families frequently prioritize maintaining optimism over full disclosure [22, 26]. Across categories, the majority of respondents (89.4%) supported disclosure of serious health changes and general health updates (77.9%), indicating a basic expectation of being kept informed. However, only 64.8% favored notifying patients about the lack of specific anti-cancer therapy options, with physicians once again leading the way (88.8% transparency). This might reflect cultural concerns in Saudi Arabia, where revealing treatment limits can be seen as reducing hope or faith in medical care.

Similar observations have been reported in other Muslim-majority, non-Arab countries. Studies from Turkey and Malaysia indicate that cancer disclosure practices are influenced not only by shared Islamic ethical values but also by country-specific cultural traditions, family roles, and healthcare system norms [27, 28]. These findings suggest that disclosure preferences should be interpreted within the broader sociocultural context rather than being attributed solely to religion. Table 3 shows significant variability in opinions and beliefs about patient engagement and information sharing. More than half of cancer patients (51.5%) and over three-quarters of healthcare practitioners (73.1%) opposed withholding information at the request of family members, while fewer than half of the general population (46.8%) agreed. This disparity highlights the cultural contradiction between family-centered decision-making, which remains strongly embedded in Middle Eastern society, and the growing emphasis on patient autonomy in medical ethics [22, 26].

Support for patient involvement in treatment discussions was high across all groups (66.3% of patients, 76.5% of the general public, and 84.3% of providers), consistent with international evidence that shared decision-making improves patient satisfaction and treatment adherence [2, 9]. Patients’ somewhat lower endorsement levels may reflect the persistence of internalized cultural norms regarding family or physicians. Patients agreed the most on end-of-life conversations (80.7%), followed by the general public (74.8%) and clinicians (67.2%) [29]. This discovery is interesting since it undermines long-held beliefs that patients in Middle Eastern settings prefer not to discuss death and dying. Instead, it reflects a trend toward greater transparency and engagement in end-of-life decisions, particularly among patients themselves [30, 31].

Participants’ expectations for the sharing of bad cancer news. Across all categories, the patient was the favored initial recipient, followed by a spouse or close member. This conclusion suggests increasing acceptability of direct patient disclosure, which is consistent with international ethical norms and aligns with trends observed in previous Saudi and regional research [14, 15, 32]. However, the persistent priority of spouses and relatives as secondary beneficiaries demonstrates the long-standing importance of family engagement in decision-making in Middle Eastern societies. When asked who they wanted to bring cancer-related news to, most respondents chose the medical team leader, followed by a team member or, less commonly, a social worker. This indicates a strong reliance on senior physicians’ authority and trustworthiness, which is consistent with the hierarchical medical traditions in many non-Western healthcare systems.

In terms of disclosure technique, many participants favored beginning with a religious framing (“Allah’s will”) or a planned introduction to the illness, while only a minority supported delivering the news straight without preparation. This study demonstrates how Islamic beliefs and cultural sensitivity influence communication choices and supports previous research that emphasizes the importance of religious and contextual framing in reducing distress during disclosure [22, 24]. Our findings are also consistent with previous studies from Riyadh and Makkah, which reported a preference for direct disclosure of cancer diagnoses while recognizing the continued importance of family involvement in communication and decision-making [15, 20]. This suggests broadly similar disclosure preferences across different regions of Saudi Arabia. Taken together, our data highlight that, while Saudi patients and families increasingly support direct, face-to-face disclosure by senior clinicians, cultural expectations such as religious framing and family engagement remain important components of the process. To fulfill the demands of a wide range of stakeholders, effective gynecologic oncology communication methods must incorporate ethical standards, cultural values, and patient-focused care.

## Conclusions

This mixed-methods study focuses on the complex dynamics underlying the disclosure of a gynecological cancer diagnosis in Saudi Arabia. While the majority of participants favored direct disclosure, significant disparities appeared regarding prognosis, treatment failure, and end-of-life discussions. These variances highlight the role of cultural norms, family participation, and professional practices in shaping communication preferences and views. Importantly, patients reported a strong desire to be educated and actively involved in treatment and end-of-life decisions, indicating a trend towards patient-centered care within an Islamic cultural context. Efforts to standardize disclosure methods must strike a balance between worldwide ethical standards and local cultural norms, while upholding transparency, patient autonomy, and respect for family responsibilities. Future studies should examine culturally responsive frameworks to help both patients and families navigate the cancer care process.

## Data Availability

The data generated and analyzed in this study are available from the corresponding author upon reasonable request.
